# Supplementary material for: Resolving the full spectrum of human genome variation using Linked-Reads
Source: Genome Res. 2019 Apr;29(4):635–45. doi: 10.1101/gr.234443.118 (PMC6442396; doi:10.1101/gr.234443.118)
Supplement: Supplemental Material [file supp_gr.234443.118_Supplemental_longranger-2.2.zip › longranger-2.2.tar.bz2!/lr2.2-public/lib/stan/cmdstan/stan/doc/api/html/math_2functions_2owens__t_8hpp_source.html]

Stan: src/stan/math/functions/owens\_t.hpp Source File


|  |  |
| --- | --- |
|  | Stan  2.5.0  probability, sampling & optimization |


- Main Page
- Related Pages
- Namespaces
- Classes
- Files

- File List
- File Members

All Classes Namespaces Files Functions Variables Typedefs Enumerations Enumerator Friends Macros Pages

- src
- stan
- math
- functions

owens\_t.hpp

Go to the documentation of this file.

1 #ifndef STAN\_\_MATH\_\_FUNCTIONS\_\_OWENS\_\_T\_HPP

2 #define STAN\_\_MATH\_\_FUNCTIONS\_\_OWENS\_\_T\_HPP

3

4 #include <boost/math/tools/promotion.hpp>

5 #include <boost/math/special\_functions/owens\_t.hpp>

6

7 namespace stan {

8  namespace math {

9

10

62  template <typename T1, typename T2>

63  inline typename boost::math::tools::promote\_args<T1,T2>::type

64  owens\_t(const T1& h, const T2& a) {

65  return boost::math::owens\_t(h, a);

66  }

67  }

68 }

69

70 #endif

stan::math::owens\_t

boost::math::tools::promote\_args< T1, T2 >::type owens\_t(const T1 &h, const T2 &a)

The Owen's T function of h and a.

**Definition:** owens\_t.hpp:64


---

[ Stan Home Page ]

*© 2011–2014,
Stan Development Team.*
